# Priority effects in a planktonic bloom-forming marine diatom

**DOI:** 10.1098/rsbl.2015.0184

**Published:** 2015-05

**Authors:** Josefin Sefbom, Ingrid Sassenhagen, Karin Rengefors, Anna Godhe

**Affiliations:** 1 Department of Biological and Environmental Sciences, University of Gothenburg, Gothenburg, Sweden; 2 Aquatic Ecology, Department of Biology, Lund University, Lund, Sweden

**Keywords:** priority effects, intraspecific competition, diatoms

## Abstract

Priority effects occur when a species or genotype with earlier arrival has an advantage such that its relative abundance in the community or population is increased compared with later-arriving species. Few studies have dealt with this concept in the context of within-species competition. *Skeletonema marinoi* is a marine diatom that shows a high degree of genetic differentiation between populations over small geographical distances. To test whether historical events such as priority effects may have been important in inducing these patterns of population differentiation, we performed microcosm experiments with successive inoculation of different *S. marinoi* strains. Our results show that even in the absence of a numerical advantage, significant priority effects were evident. We propose that priority effects may be an important mechanism in initiating population genetic differentiation.

## 1. Introduction

Priority effects occur when a species or genotype with early arrival to a vacant resource gains an advantage [1] resulting in an increased relative abundance in the community compared with later-arriving species or genotype. This concept has been studied rigorously in the context of interspecific interactions in community assembly, biological invasions and restoration ecology [2,3]. However, only a limited number of studies have investigated the importance of priority effects at the intraspecific level, e.g. in amphibians, fish and bacteria [4–8]. In these studies, it was shown that the timing of arrival significantly affected the competitive strength [6,7], diversification [8] and even mortality within the same species [4,5].

Microorganisms such as phytoplankton are argued to have a ubiquitous dispersal, as an effect of their small size and immense population sizes [9]. Yet, a growing number of studies on phytoplankton population genetics provide results indicative of low gene flow among populations sampled less than 100 km apart [10–12]. In light of this conflict of low gene flow despite high dispersal potential [13,14], we wanted to investigate the importance of intraspecific priority effects in segregating adjacent populations of planktonic phytoplankton.

We experimentally examined intraspecific priority effects in *Skeletonema marinoi*, a species displaying low genetic connectivity across small spatial scales [11]. *Skeletonema marinoi* is a centric marine diatom that has a wide global distribution and is common during the spring bloom in temperate regions [15]. We hypothesized that early arrival of a strain increases its relative abundance in the population compared with arriving later. To test this hypothesis, we performed microcosm competition experiments with cultured strains of *S. marinoi*. In a natural scenario, new invaders may continuously arrive, environmental changes are erratic and local adaptation may provide an additional advantage [14]. Here, we have excluded these factors, including any numerical advantage of the early-arriving strains, in order to exclusively test the influence of successive arrival in the competition between strains.

## 2. Material and methods

Three strains were used in this study: Lys6D (A), Lys6S (B) and St31 (C), which were supplied by Gothenburg University Marine Algal Culture Collection. The strains had previously been genotyped using eight microsatellite markers (S.mar1–8) [16]. Cultures were grown in 26 PSU f/2 medium [17], at 10°C and a light : dark cycle of 12 : 12 h (irradiance 50 μmol photons s^−1^ m^−2^). Conditions remained unchanged during all experiments. All culturing was carried out in 200 ml Nunc flasks (three replicates) with starting concentrations of 5000 cells ml^−1^ per strain. Cell counts were performed daily, with a Sedgewick rafter-counting chamber using an inverted microscope (Axiovert 135, Zeiss).

A growth study was performed to characterize maximum growth rates of the experimental strains. Maximum growth rates were calculated as: *μ*_max_ = ln(*N*_2_/*N*_1_)/(*t*_2_ − *t*_1_). To correct for possible cell-count errors, we used a sliding window where data points were taken on 3-day intervals [18]. Differences between strains were tested with a one-way ANOVA and corrected with Bonferroni adjustment (IBM SPSS Statistics v. 22).

Priority experiments were conducted in all possible combinations of two strains, either with concurrent inoculation (control) or with a time lag (priority treatments). All strains were tested with early arrival (founder) and later arrival (invader). Growth was monitored daily throughout the experiments. The invader strain was added when the founder strain had initiated exponential growth, which was after 3 days. To ensure actively growing invader strains, their cultures had been enriched with fresh medium 3 days prior to inoculation. The invader strain was added at equal density to the founder at the time of inoculation (strain ratio 1 : 1). This set-up circumvented a numerical advantage for the founding strain. Experiments were terminated in the early stationary phase, which was after 10 days in experiment A/B, and after 9 days for experiments A/C and B/C (electronic supplementary material, S1). All experimental bicultures were filtered onto separate 3.0 µm filters (Versapore®- 3000T, Pall Cooperation) and stored at −80°C.

Genomic DNA was extracted from the filters using the cetyl trimethylammonium bromide (CTAB) method [19]. Three microsatellite loci (S.mar1, S.mar5 and S.mar6) [16] were subsequently amplified with PCR conditions described by Godhe & Härnström [11]. To assess the relative abundance of each strain in the bicultures, we used an allele-specific quantitative PCR (AsQ-PCR) method [20] (electronic supplementary material, S2). Statistical testing for priority effects was done with a one-way ANOVA on relative proportions of strains (arcsine of square root transformed) and Dunnett's *post hoc* (IBM SPSS Statistics v. 22).

Growth rates for each strain in bicultures were calculated (see electronic supplementary material, S3) and tested using a two-way ANOVA with Bonferroni correction.

## 3. Results

The growth study on monocultures lasted 9 days (electronic supplementary material, S4). Highest maximum growth rate was measured in strain A (0.76 divisions d^−1^), then C and B (0.70 and 0.69 divisions d^−1^, respectively). Statistical analysis showed no significant difference between strains (*F*_2,6_ = 2.054, *p* > 0.1).

**Figure 1.**
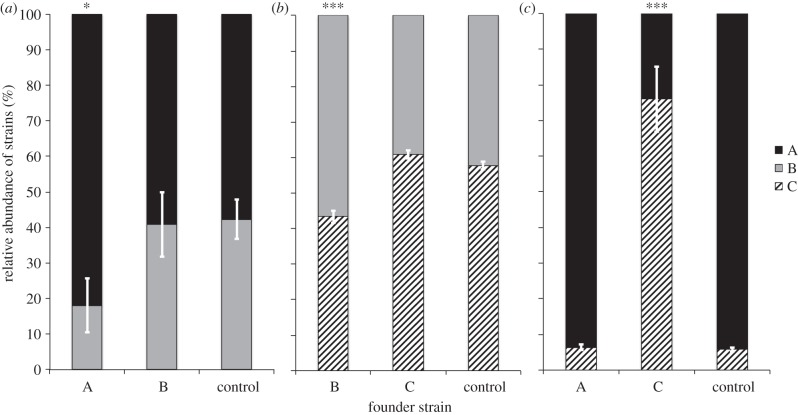
(*a–c*) Relative abundances of respective *S. marinoi* strains in mixed cultures determined by microsatellite marker peak-height ratios (AsQ-PCR). Founder strain grew for 3 days before invading strain was added. Reciprocal priority treatments and control between strain-pair: (*a*) A and B; (*b*) B and C; (*c*) A and C. Error bars indicate standard deviation of the mean (*n* = 3). Asterisks indicate significant differences in relative abundance compared with the control (*p < 0.05; ***p < 0.001).

In the priority effects experiments, relative abundances of strains showed statistically significant priority effects (A/B: *F*_2,6_ = 9.964, *p* < 0.01, B/C: *F*_2,6_ = 137.8, *p* < 0.001, A/C: *F*_2,6_ = 159.4, *p* < 0.001). *Post hoc* analyses revealed that one strain in each experiment increased significantly compared with the control. For strains A/B, A dominated in the control (57.8 ± 5.5%; mean ± s.d.) and both treatments (figure 1*a*). When A was inoculated first it significantly increased to 82% ± 7.6% (*p* = 0.013). When the competing strain B was given priority, no significant increase could be seen. For B/C (figure 1*b*), strain C surpassed B in the control (57.8 ± 1%), but B gained an advantage through priority (from 42.2% ± 1% to 56.6 ± 1.8%; *p* < 0.001). Strain C increased by 3% when inoculated first, but this was not statistically significant. In set A/C (figure 1*c*), strain A dominated in the control, but C showed a significant advantage from priority, increasing from 5.8% ± 0.6% to 76 ± 9.2% (*p* < 0.001).

**Figure 2.**
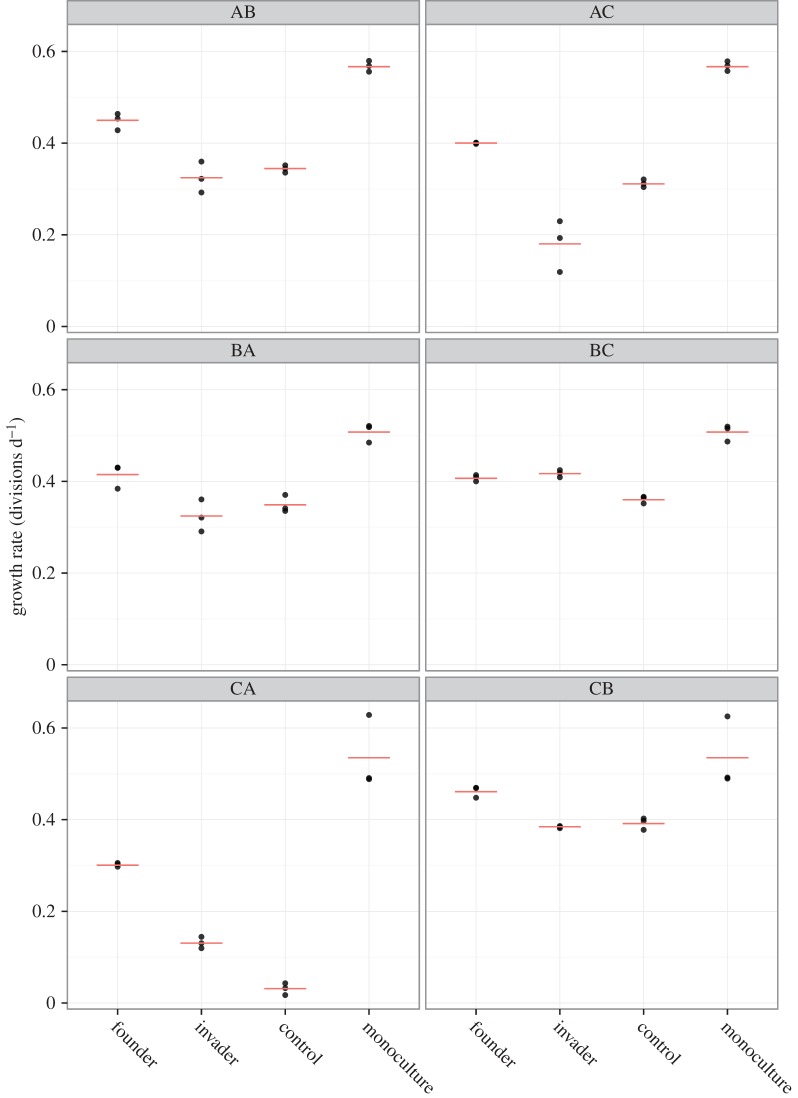
Strain-specific growth rates in each experiment and monoculture. The first letter in each header (XX) indicates the measured strain and the second letter (XX) indicates the competing strain. Raw data are symbolized as dots and averages as horizontal lines (*n* = 3). (Online version in colour.)

Comparisons of strain-specific growth rates between treatments revealed significant changes (*F*_3,2_ = 16.411 *p* < 0.001) in all strains (figure 2). *Post hoc* analysis showed that there was a significant negative effect on growth when in biculture compared with growing in monoculture (*p* < 0.001). However, prior arrival resulted in a significantly increased growth rate compared with simultaneous or later arrival (*p* < 0.001). There was no significant difference between the latter two treatments (*p* > 0.05).

## 4. Discussion

This study supports the hypothesis that early arrival of a strain increases its relative abundance compared with simultaneous or later arrival, even when numerical effects are excluded. Prior arrival showed either a positive or neutral effect on the competitive ability, depending on the strain. In addition, we show that there was an overall negative effect on growth rates when in biculture compared with monocultures, but that first arrival significantly mitigates this negative effect.

In the absence of priority effects, competitive strength in bicultures reflected fitness as measured by maximum growth rate in monocultures. This was observed in treatments with simultaneous inoculation of both strains, where the fastest-growing strain in monoculture dominated. Whereas, prior arrival increased growth rates in all founder strains and in three out of six times this resulted in a significantly increased relative abundance.

Our findings concur with those reported for the planktonic cyanobacterium *Microcystis* [7]. However, in our study, priority effects were already apparent with only 3 days between inoculations (instead of one week), and without a numerical advantage of the founder strain. Similar to our results, significant priority effects were only found for one *Microcystis* strain in each combination of strains. Therefore, both our results and those for *Microcystis* indicate a strong strain-dependent effect size. The high degree of variation in growth rates displayed within each strain when growing together with different strains supports that there are strain-specific interactions occurring. A possible explanation for the strain-specific priority effects is the production of polyunsaturated aldehydes (PUAs) in *S. marinoi*, which has been shown to vary between strains [21]. These PUAs are associated with inhibitory growth effects on phytoplankton species, including *S. marinoi* [22].

Our experiments show that prior arrival can alter the intrinsic competitive abilities of phytoplankton strains. In the absence of priority effects, competitive strength appears largely influenced by intrinsic growth rates as observed in monocultures, whereas prior arrival adds a beneficial element in the competition between strains. Strikingly, we observed these effects in the absence of numerical advantages. To the best of our knowledge, intraspecific priority effects have never previously been reported in planktonic protists. Based on our results, we suggest that priority effects increase competitive ability of early-arriving strains and restricts establishment of later-arriving genotypes. This may contribute to the genetic differentiation that is observed among phytoplankton populations. In fast-growing organisms such as diatoms, we could expect that priority effects be further reinforced by a numerical advantage. Over time, genetic adaptation in response to local environmental conditions can serve as an additional stabilizing mechanism that promotes genetic differentiation [14]. Our observations on priority effects likely also apply to other planktonic protists that demonstrate similar population genetic patterns.

## Supplementary Material

Suppl1_ExpGrowthCurves

## Supplementary Material

Suppl2_AsQ-PCR

## Supplementary Material

Suppl3_growth-calc

## Supplementary Material

Supple4_MonoGrowth

## Data accessibility

The datasets supporting this article can be accessed in Dryad (http://dx.doi.org/10.5061/dryad.rg68j).
