# Supplementary material for: Priority effects in a planktonic bloom-forming marine diatom
Source: Biol Lett. 2015 May;11(5):20150184. doi: 10.1098/rsbl.2015.0184 (PMC4455744; doi:10.1098/rsbl.2015.0184)
Supplement: Suppl1_ExpGrowthCurves [file rsbl20150184supp1.docx]

**Supplement 1 (S1)**

**Figure S1a-c.** Growth curves from the three experimental sets. Error bars indicate the standard deviation of the mean (n=3). a) Priority treatments of strains A (then B added 3 days later) and B (then A added 3 days later) and the Control (concurrent inoculation). b) Priority treatments of strains C (then B added 3 days later) and B (then C added 3 days later) and the Control (concurrent inoculation) c) Priority treatments of strains A (then C added 3 days later) and C (then A added 3 days later) and the Control (concurrent inoculation).
