# Supplementary material for: Priority effects in a planktonic bloom-forming marine diatom
Source: Biol Lett. 2015 May;11(5):20150184. doi: 10.1098/rsbl.2015.0184 (PMC4455744; doi:10.1098/rsbl.2015.0184)
Supplement: Suppl2_AsQ-PCR [file rsbl20150184supp2.docx]

**Supplement 2 (S2)**

**Allele-specific quantitative-PCR**

Respective peak-heights in the electropherograms were used as a relative quantification measurement. To ascertain non-biased PCR amplification the strain combinations were mixed in eight known proportions ranging from 5:95 to 80:20 (3 replicates) and collected on filters. DNA was extracted using a CTAB based protocol [[1](#_ENREF_1)]. DNA concentration and purity was measured on a Nanodrop 2000C (Thermo Scientific). Three microsatellite loci were amplified (S.mar1, S.mar5 and S.mar6[[2](#_ENREF_2)]) by polymerase chain reactions (PCR) using conditions described in [Godhe and Härnström [3](#_ENREF_3)]. These three loci were chosen (out of the eight available) as all strains could easily be identified based on the respective fragment lengths of these markers. The products were analyzed in an ABI 3730 (Applied Biosystems) and allele sizes were assigned relative to the internal standard GS600LIZ. Genotyping was determined using GeneMapper (ABI Prism®GeneMapper™Software Version 3.0). To evaluate non-bias PCR reactions peak-height relative abundances were plotted against known relative abundances to obtain *r^2^*-values.

Locus S.mar 6 showed little to no bias in the PCR reaction for all three strain combinations and proportions. All respective alleles were represented in the electropherograms. Relative peak-heights plotted against known relative abundances (Figure S1a-c) gave *r^2^*-values of 0.989, 0.993, and 0.983 for the respective strain pairs A/B, B/C and A/C.

**Figure S 2a-b**. The relative abundance of one strain, in a two-strain mix, determined by cell count and on the x-axis, and proportional peak heights of respective strains in the electropherogram after fragment amplification on the y-axis. a) Strain A in proportion to B; b) strain A in proportion to C; c) strain B in proportion to C. Error bars indicate standard deviation of the mean (n=3).

**References**

1. Kooistra W., De Stefano M., Mann D.G., Salma N., Medlin L.K. 2003 Phylogenetic position of Toxarium, a pennate-like lineage within centric diatoms (Bacillariophyceae). *J Phycol* **39**(1), 185-197. (doi:10.1046/j.1529-8817.2003.02083.x).

2. Almany G.R., De Arruda M.P., Arthofer W., Atallah Z.K., Beissinger S.R., Berumen M.L., Bogdanowicz S.M., Brown S.D., Bruford M.W., Burdine C., et al. 2009 Permanent Genetic Resources added to Molecular Ecology Resources Database 1 May 2009-31 July 2009. *Molecular Ecology Resources* **9**(6), 1460-1466. (doi:10.1111/j.1755-0998.2009.02759.x).

3. Godhe A., Härnström K. 2010 Linking the planktonic and benthic habitat: genetic structure of the marine diatom Skeletonema marinoi. *Mol Ecol* **19**(20), 4478-4490. (doi:Doi 10.1111/J.1365-294x.2010.04841.X).
