# Supplementary material for: Priority effects in a planktonic bloom-forming marine diatom
Source: Biol Lett. 2015 May;11(5):20150184. doi: 10.1098/rsbl.2015.0184 (PMC4455744; doi:10.1098/rsbl.2015.0184)
Supplement: Suppl3_growth-calc [file rsbl20150184supp3.docx]

**Supplement 3 (S3)**

**Calculation of growth rates in biculture**

Growth rates for each strain in bicultures and monoculture were calculated as:

g=(1/*T*) * Ln(D*_T_*/D*_0_*)

Where *T* is days from inoculation of each strains to experimental termination, D*_T_* is the cell density at the end of the experiments calculated as the proportion (established with AsQ-PCR) of the final cell density. D*_0_* is the starting cell density for each separate inoculate (i.e. 5000 cells mL^-1^ for founder strains and control treatments, for invader strains >5000 cells mL^-1^).
