# Supplementary material for: Priority effects in a planktonic bloom-forming marine diatom
Source: Biol Lett. 2015 May;11(5):20150184. doi: 10.1098/rsbl.2015.0184 (PMC4455744; doi:10.1098/rsbl.2015.0184)
Supplement: Supple4_MonoGrowth [file rsbl20150184supp4.docx]

**Supplement 4 (S4)**

**Figure S4.** Growth curves for strains A, B and C in monoculture. Error bars indicate standard deviation of the mean (n=3). Maximum growth rates were 0.76, 0.69 and 0.70 divisions day^-1^ for strains A, B and C, respectively. No significant differences between strains (*F_2,6_*=2.054, *p*>0.1).
